# Adipose Tissue Adaptations to Chronic Kumquat Consumption in High-Fat Diet-Induced Obese Mice

**DOI:** 10.3390/cimb48101048

**Published:** 2026-10-10

**Authors:** Simona Terzo, Pasquale Calvi, Marta Giardina, Salvatore Timeo, Assunta Lombardi, Antonella Amato, Flavia Mulè

**Affiliations:** 1 Department of Biological, Chemical, Pharmaceutical Science and Technology (STEBICEF), University of Palermo, 90128 Palermo, Italy; simona.terzo01@unipa.it (S.T.); pasquale.calvi@unipa.it (P.C.); marta.giardina@unipa.it (M.G.); salvatore.timeo@community.unipa.it (S.T.); antonella.amato@unipa.it (A.A.); 2 Dipartimento di Biologia, Università degli Studi di Napoli Federico II, Monte Sant’Angelo, 80126 Naples, Italy; aslombar@unina.it

**Keywords:** adiposity, inflammation, kumquat, thermogenesis, high fat diet

## Abstract

White adipose tissue dysfunction is a central event in the development of obesity-associated metabolic disorders. Kumquat (*Fortunella japonica*) is consumed whole, allowing intake of peel-derived bioactive compounds. Although increasing evidence suggests that kumquat exerts beneficial metabolic effects, its impact on white adipose tissue homeostasis remains poorly understood. We investigated whether chronic supplementation with lyophilized whole kumquat could attenuate adipose tissue dysfunction in high-fat diet (HFD)-fed mice. Male C57BL/6J mice received a standard diet, HFD, or HFD supplemented with 5% (*w*/*w*) kumquat (HFD + K) for 24 weeks. Kumquat attenuated body weight gain, reduced food intake and caloric efficiency, and decreased epididymal white adipose tissue mass. Histological analysis showed reduced adipocyte hypertrophy and a shift toward smaller adipocytes. Kumquat also decreased macrophage crown-like structures and *Tnf* and *Il1b* expression, while increasing circulating adiponectin, lowering leptin, and improving the leptin/adiponectin ratio. At the molecular level, kumquat increased *Pparg*, *Ppargc1a*, *Sirt1*, *Cox4i1*, and *Ucp1* expression, together with mitochondrial DNA copy number, consistent with changes in molecular markers associated with mitochondrial remodelling and thermogenic pathways. Overall, these findings identify white adipose tissue as a relevant target of chronic whole-kumquat consumption during diet-induced obesity and suggest that preservation of adipose tissue homeostasis involves coordinated anti-inflammatory, endocrine, and mitochondrial-related molecular adaptations.

## 1. Introduction

Obesity has become one of the most critical public health challenges worldwide, with its prevalence steadily increasing across all age groups over the past decades. According to recent estimates, obesity affects more than one billion individuals globally and represents a major risk factor for the development of numerous chronic diseases, including type 2 diabetes mellitus (T2DM), cardiovascular diseases, metabolic dysfunction-associated steatotic liver disease (MASLD), several types of cancer, and neurodegenerative disorders [1,2,3]. The pathogenesis of obesity is highly complex and involves the interaction of genetic, environmental, behavioural, and metabolic factors that ultimately disrupt energy homeostasis and promote excessive adipose tissue accumulation. Among the mechanisms linking obesity to metabolic disease, the expansion and dysfunction of white adipose tissue (WAT) play a central role [4]. Beyond serving as the primary site for energy storage, WAT functions as a dynamic endocrine organ that regulates systemic energy homeostasis through the secretion of adipokines, cytokines, lipid mediators, and other bioactive molecules involved in glucose and lipid metabolism, insulin sensitivity, inflammation, and energy expenditure [5]. Chronic caloric excess promotes adipocyte hypertrophy and maladaptive adipose tissue remodelling, characterized by fibrosis, hypoxia, mitochondrial dysfunction, immune cell infiltration, and chronic low-grade inflammation [4,6]. These alterations are accompanied by a shift toward a pro-inflammatory secretory profile, with increased production of tumour necrosis factor-α (TNF-α), interleukin-6 (IL-6), and monocyte chemoattractant protein-1 (MCP-1), together with reduced adiponectin secretion, ultimately contributing to systemic insulin resistance, dyslipidaemia, and metabolic syndrome. Adipose tissue plasticity is increasingly recognized as a key determinant of metabolic health. Under physiological conditions, adipose tissue expands through adipogenesis and hyperplasia, allowing excess nutrients to be safely stored. Conversely, impaired adipogenic capacity promotes adipocyte hypertrophy and ectopic lipid deposition in metabolically active organs, including the liver, skeletal muscle, and pancreas, thereby exacerbating obesity-associated metabolic dysfunction [4]. Consequently, preserving adipose tissue function and remodelling capacity has emerged as a promising therapeutic target. In this context, nutritional interventions and dietary bioactive compounds have attracted considerable interest as non-pharmacological strategies to preserve adipose tissue homeostasis. Polyphenol-rich functional foods have been shown to modulate adipogenesis, attenuate inflammation and oxidative stress, improve mitochondrial function, and enhance metabolic flexibility [7,8,9,10,11]. Accordingly, several experimental studies have demonstrated beneficial effects of natural bioactive compounds on obesity-associated adipose tissue dysfunction and metabolic alterations [7,8,9,10,11,12,13], supporting their potential as complementary strategies to mitigate the metabolic consequences of obesity. Therefore, identifying novel dietary strategies capable of restoring adipose tissue homeostasis may represent an effective approach to counteract the metabolic consequences of obesity and improve cardiometabolic health.

Kumquat (*Fortunella japonica*) is a small citrus fruit traditionally consumed together with its peel, resulting in a particularly high intake of bioactive phytochemicals [14,15]. Kumquat is rich in phenolic constituents, particularly flavonoids and other reducing compounds, which could be responsible for its beneficial health-related properties [15]. Despite its rich phytochemical profile, the metabolic effects of chronic kumquat consumption have only recently begun to be explored. Evidence from animal studies indicates that supplementation with whole kumquat fruit powder or its ethanolic extract improves glycaemic control in diabetic rats and obese mice, respectively [16,17]. Moreover, our previous studies demonstrated that long-term dietary supplementation with kumquat effectively protects against high-fat diet (HFD)-induced metabolic dysfunction. Specifically, chronic kumquat intake attenuated the development of MASLD, reducing hepatic lipid accumulation, inflammation, and insulin resistance while enhancing mitochondrial β-oxidation through activation of the AMPK–PGC-1α signalling pathway [18]. In addition, kumquat supplementation exerted significant neuroprotective effects in HFD-fed mice, restoring brain insulin signalling, reducing neuroinflammation, and preventing neuronal apoptosis [19]. These findings suggest that kumquat may exert systemic metabolic benefits that extend beyond individual organs and involve common mechanisms related to the regulation of energy homeostasis. 

Given the central role of adipose tissue dysfunction in obesity-related metabolic disorders, it is plausible that adipose tissue represents a primary target of kumquat action. However, the effects of chronic kumquat consumption on adiposity, adipose tissue remodelling, and thermogenic signalling remain largely unknown. Therefore, the present study investigated whether long-term kumquat supplementation could counteract HFD-induced adiposity and adipose tissue dysfunction in mice. Notably, the present study focused on exploring the potential benefits of whole kumquat fruit, highlighting its potential role as a functional food whose nutritional and phytochemical composition has been well documented in previous studies [15,20]. Particular attention was devoted to molecular markers associated with lipid metabolism, mitochondrial remodelling, and thermogenic pathways, with the aim of elucidating the mechanisms underlying the potential anti-obesogenic effects of kumquat.

## 2. Materials and Methods

### 2.1. Animals and Diets

Four-week-old male C57BL/6J mice were purchased from Envigo (San Pietro al Natisone, Udine, Italy). Animals were housed (two mice per cage) under controlled environmental conditions, including a 12-h light/12-h dark cycle, a temperature of 22 ± 1 °C, and a relative humidity of 60 ± 5%, with ad libitum access to food and water. Following a one-week acclimatization period, mice were randomly allocated to three experimental groups (*n* = 8 per group): a standard diet group (STD; 10% of total energy from fat; 4RF25, Mucedola, Milan, Italy), a high-fat diet group (HFD; 60% of total energy from fat; PF4215, Mucedola, Milan, Italy), and a high-fat diet supplemented with 5% (*w*/*w*) lyophilized whole kumquat fruit (HFD + K). Whole kumquat fruit, including both peel and pulp, was used after lyophilization and incorporated into the diet as a powder. All diets were administered for 24 weeks. In the HFD + K formulation, 50 g/kg of sucrose was replaced with 50 g/kg of lyophilized whole-kumquat powder, corresponding to 5% (*w*/*w*) of the diet. The complete composition and energy densities of the HFD and HFD + K diets are reported in Table S1. The kumquat dose was established according to previously published studies [18,19]. The nutritional and phytochemical composition of kumquat fruit used in this study (*F. japonica*) has been extensively characterized in the literature [21,22,23,24]. Published data on the nutritional and phytochemical composition of *Fortunella japonica* are summarized in Supplementary Table S2. The animals analysed in the present study belonged to the same experimental cohort used in our previously published studies investigating the effects of chronic kumquat supplementation on the liver and brain [18,19]. The present study specifically focuses on adipose tissue outcomes that were not addressed in those previous investigations. Body weight was recorded weekly throughout the experimental period. Food intake was measured weekly at the cage level (two mice per cage; four cages per experimental group) and expressed as the average food intake per mouse. For statistical analysis of food intake, the cage was considered the experimental unit (*n* = 4 cages per group). Caloric efficiency was determined by calculating the ratio between the change in body weight and the total amount of energy consumed (kcal ingested) over the experimental period. After 24 weeks, mice were subjected to an overnight fast and then euthanized. Blood was collected by cardiac puncture into EDTA-containing tubes. The epididymal white adipose tissue (eWAT) was excised, weighed, and either stored at −80 °C or fixed in 10% formalin for subsequent analyses. All experimental procedures involving animals were conducted in accordance with the Italian Legislative Decree No. 26/2014 and were approved by the Italian Ministry of Health (Rome, Italy; Authorization No. 46/2020-PR, issued on 21 January 2020).

### 2.2. Histology and Immunohistochemistry

For morphological examination, eWAT was fixed in 10% buffered formalin for 24 h, dehydrated in alcohol, and embedded in paraffin. Histological sections (5 µm thick) were stained with haematoxylin and eosin and observed using an optical microscope (Leica DMLB, Meyer Instruments, Houston, TX, USA) connected to a high-resolution camera (DS-Fi1, Nikon, Florence, Italy). Adipocyte size was assessed by measuring cell diameter [25]. For each animal, two adipose tissue sections were analysed, with four microscopic fields examined per section. Morphometric analysis was performed at 20× magnification on 50 adipocytes per animal. Histological analyses were performed by two investigators blinded to the experimental group allocation. For adipocyte-size distribution analysis, the individual area measurements obtained from these 50 adipocytes per mouse were grouped into predefined 200 µm^2^ size intervals and the frequency of adipocytes within each size class was expressed as a percentage of the total number of adipocytes analysed. For immunohistochemical analysis, adipose tissue samples were first deparaffinized using xylene, followed by suppression of endogenous peroxidase activity through incubation with 3% hydrogen peroxide for 30 min at room temperature. After washing with PBS, tissue sections were incubated overnight with the primary Mac-2 antibody (1:2800 dilution; Cedarlane, Burlington, ON, Canada, Cat. No. CL8942AP). Subsequently, sections were rinsed three times with PBS and exposed to a biotin-conjugated secondary antibody (Anti-Mouse IgG/Rabbit IgG; 1:400 dilution; Vector Laboratories, Burlingame, CA, USA, Cat. No. BA-4001) for 30 min at room temperature. The staining procedure was completed using the ABC reagent from the Elite ABC kit (Vector Laboratories, Burlingame, CA, USA) and diaminobenzidine (Sigma, Milan, Italy), following the manufacturers’ instructions. Crown-like structures (CLSs) were identified and counted, and the results were reported as the number of CLS per 10,000 adipocytes.

### 2.3. Plasma Hormone Measurements

Plasma levels of glucagon-like peptide-1 (GLP-1), ghrelin, adiponectin, and leptin were measured using commercially available enzyme-linked immunosorbent assay (ELISA) kits according to the manufacturers’ instructions. The following kits were used: Mouse GLP-1 ELISA Kit (A75436, Antibodies.com, Cambridge, UK), Mouse Ghrelin ELISA Kit (A2420, Antibodies.com, Cambridge, UK), ELISA Kit for Adiponectin (ADPN) (SEA605Mu, Cloud-Clone Corp., Katy, TX, USA), and ELISA Kit for Leptin (LEP) (SEA084Mu, Cloud-Clone Corp., Katy, TX, USA). Blood samples were collected in appropriate tubes and centrifuged at 3000 rpm for 15 min at 4 °C to obtain plasma. Plasma aliquots were stored at −80 °C until analysis. Samples were appropriately diluted when required and analysed in duplicate. Absorbance was measured at 450 nm using a BioTek 800 TS Absorbance Reader (Agilent Technologies, Santa Clara, CA, USA). Hormone concentrations were calculated from standard curves generated using the corresponding reference standards and expressed as pg/mL, ng/mL, or µg/mL, as appropriate.

### 2.4. Real-Time RT-PCR

Total RNA was extracted from eWAT using the Invitrogen™ PureLink™ RNA Mini Kit (Thermo Fisher Scientific, Waltham, MA, USA). Equal amounts of total RNA were reverse transcribed into complementary DNA (cDNA) using the Applied Biosystems™ High-Capacity cDNA Reverse Transcription Kit (Thermo Fisher Scientific, Waltham, MA, USA). Quantitative real-time PCR was performed on a QuantStudio™ 3 Real-Time PCR System (Applied Biosystems, Thermo Fisher Scientific, Waltham, MA, USA) using the Applied Biosystems™ SYBR™ Green Universal Master Mix (Thermo Fisher Scientific, Waltham, MA, USA). Gene-specific amplification was performed using QuantiTect Primer Assays (QIAGEN, Hilden, Germany), as listed in Table 1. Relative gene expression levels were calculated using the 2^−ΔΔCt^ method, with β-actin as the endogenous reference gene.

### 2.5. Mitochondrial DNA (mtDNA) Copy Number Quantification

Genomic DNA was extracted from frozen eWAT samples using the DNeasy Blood & Tissue Kit (QIAGEN, Hilden, Germany), according to the manufacturer’s instructions. Absolute mtDNA copy number was quantified using the Mouse Absolute Mitochondrial DNA Copy Number Quantification Kit (RayBiotech, Norcross, GA, USA), a TaqMan™ probe-based quantitative PCR assay. The assay specifically amplifies a conserved region of mouse mitochondrial DNA and the single-copy nuclear gene 36B4, which was used as an internal reference for normalization.

Real-time PCR reactions were performed using a QuantStudio™ 3 Real-Time PCR System (Applied Biosystems, Thermo Fisher Scientific, Waltham, MA, USA), and samples were analysed in duplicate. According to the manufacturer’s protocol, mitochondrial DNA copy number was calculated using the comparative ΔΔCq method relative to the supplied Reference Mouse DNA, which has an assigned mtDNA copy number of 2500 copies per diploid cell. Absolute mtDNA copy number was calculated as 2500 × 2^−ΔΔCq^ and expressed as mtDNA copies per diploid cell.

### 2.6. Statistical Analysis

All results are presented as the mean ± standard error of the mean (SEM). Statistical analyses were carried out using GraphPad Prism 6 (GraphPad Software, San Diego, CA, USA). For measurements obtained from individual animals, the individual mouse was considered the experimental unit (*n* = 8 per group). For food-intake measurements, which were collected at the cage level, the cage was considered the experimental unit (*n* = 4 cages per group). Differences among experimental groups were evaluated by ordinary one-way analysis of variance (ANOVA), followed by Bonferroni’s multiple-comparisons post hoc test when the assumptions for parametric analysis were met. Weekly body weight and food-intake data were analysed using two-way repeated-measures ANOVA, followed by Bonferroni’s multiple-comparisons test. A value of *p* < 0.05 was considered statistically significant.

## 3. Results

### 3.1. Changes in Body Weight Gain, in Food Intake and Caloric Efficiency

**Figure 1:**
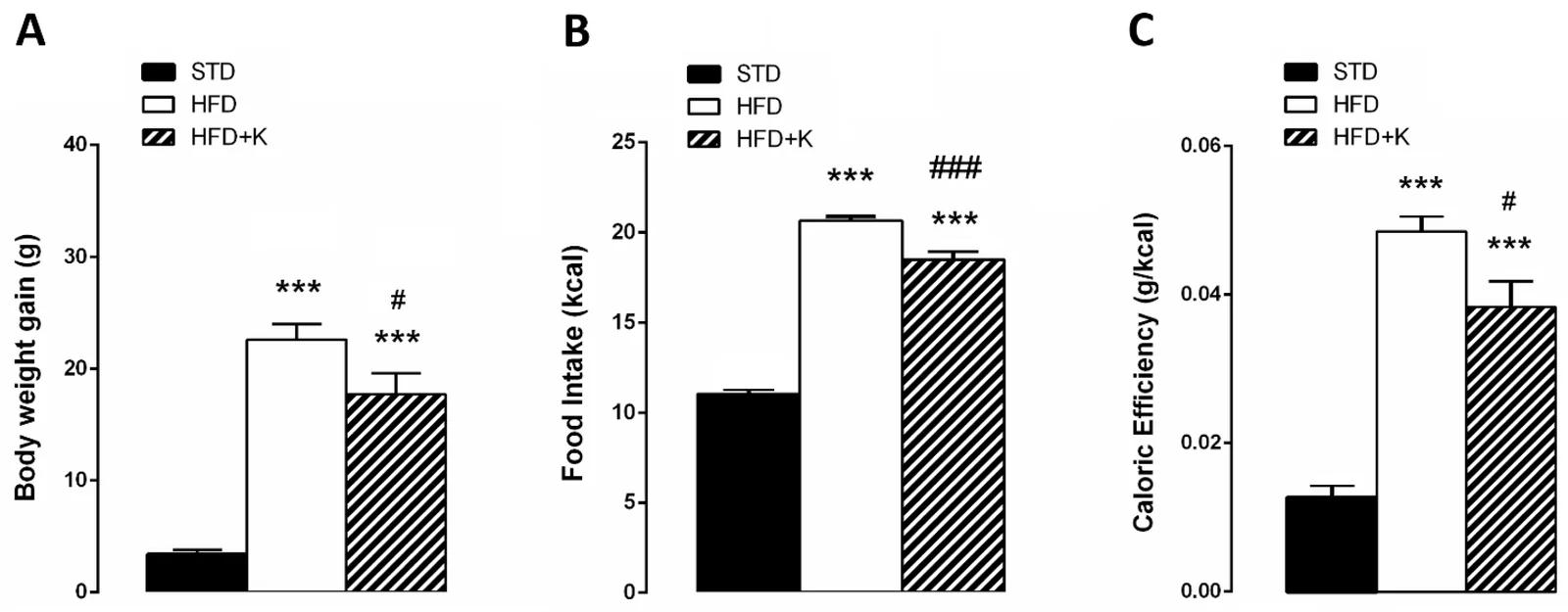
(**A**) Body weight gain, (**B**) food intake and (**C**) caloric efficiency among STD, HFD and HFD + K mice. Results are expressed as means ± SEM. For body weight gain, *n* = 8 mice per group; for food intake and caloric efficiency, *n* = 4 cages per group. *** *p* < 0.001 versus STD mice; # *p* < 0.05, ### *p* < 0.001 versus HFD mice.

At week 24, body weight gain was significantly higher in the High Fat Diet (HFD) group than in the Standard diet (STD) group, confirming the development of diet-induced obesity. Kumquat supplementation in HFD-fed mice attenuated body weight gain compared with HFD controls (Figure 1A). Food intake was higher in the HFD group than in the STD group. HFD + K mice exhibited lower food intake than HFD controls (Figure 1B). Figure S1 shows the weekly changes in body weight and food intake measured throughout the experimental period. Furthermore, caloric efficiency was significantly lower in HFD + K mice than in HFD controls (Figure 1C).

### 3.2. Changes in Metabolic Hormones

**Figure 2:**
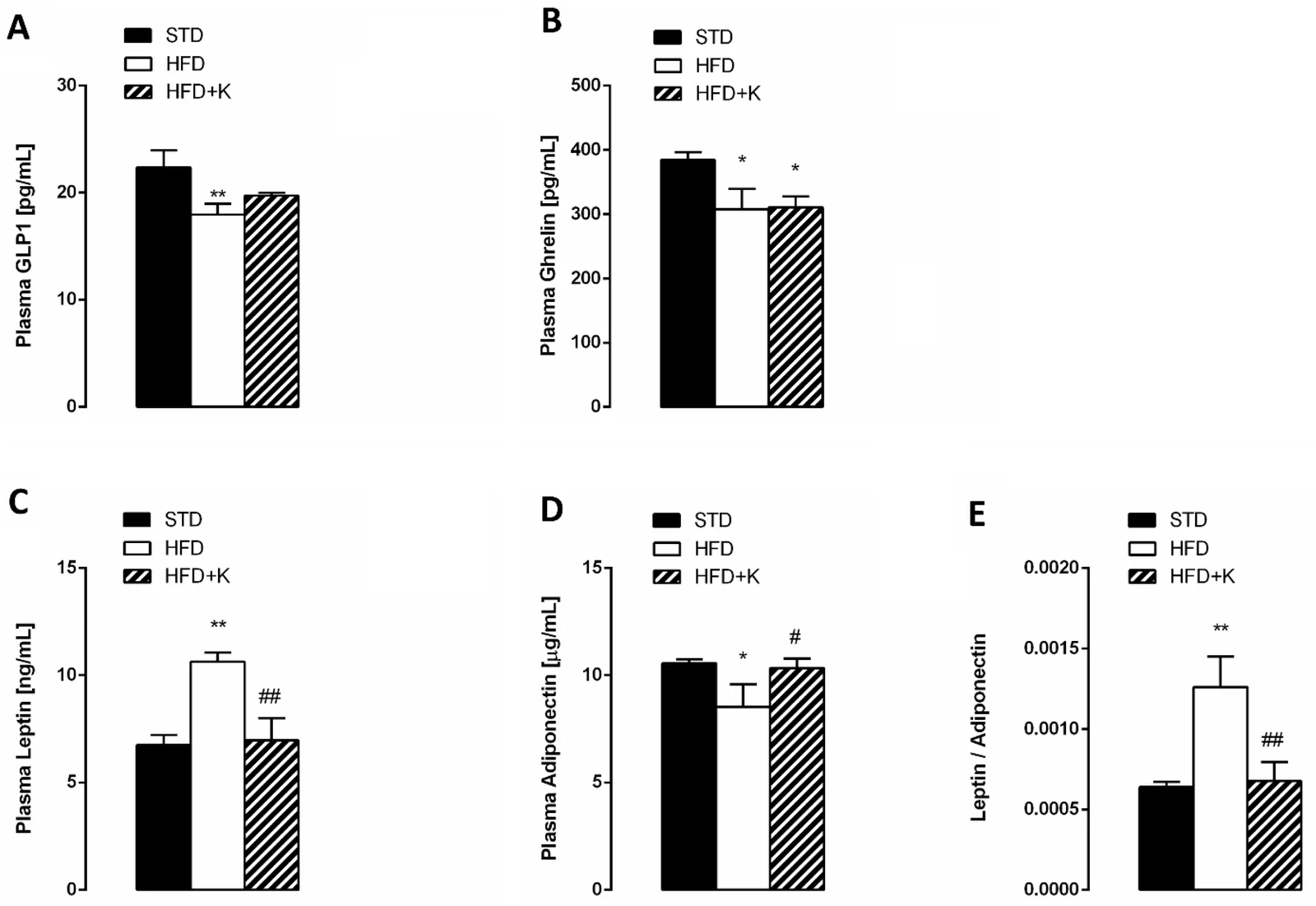
Plasma concentrations of (**A**) GLP-1, (**B**) ghrelin, (**C**) leptin, (**D**) adiponectin, and (**E**) leptin/adiponectin ratio in STD, HFD, and HFD + K mice. Results are expressed as means ± SEM (*n* = 8). * *p* < 0.05, ** *p* < 0.01 versus STD mice; # *p* < 0.05, ## *p* < 0.01 versus HFD mice.

Since HFD + K mice exhibited reduced food intake, we measured circulating levels of key metabolic hormones involved in the regulation of food intake and energy homeostasis, including glucagon-like peptide-1 (GLP-1), ghrelin, leptin, and adiponectin. Circulating GLP-1 levels were significantly reduced in both HFD and HFD + K mice compared with the STD group (Figure 2A). Similarly, ghrelin levels were decreased in HFD and HFD + K mice relative to the STD group (Figure 2B). Moreover, HFD feeding induced an increase in leptin levels and a reduction in adiponectin levels compared with the STD group. These alterations were reversed by kumquat supplementation. Consistently, the leptin/adiponectin ratio was significantly elevated in HFD mice and significantly reduced in the HFD + K group compared with HFD animals (Figure 2C–E).

### 3.3. Changes in Adiposity and Adipocyte Morphology

**Figure 3:**
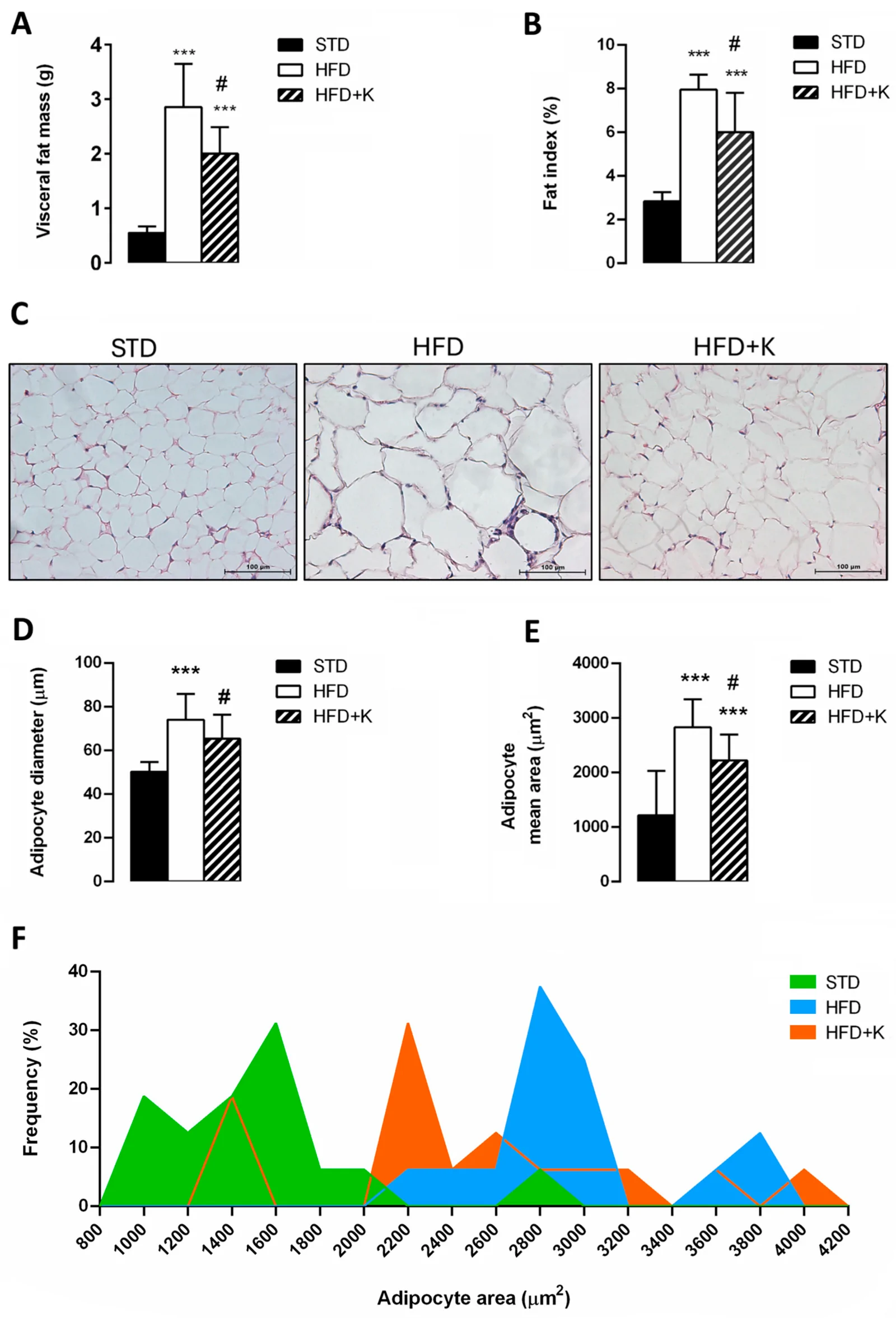
(**A**) Epididymal white adipose tissue (eWAT) weights; (**B**) eWAT weights as a percentage of body weight; (**C**) H&E staining of representative eWAT sections in different groups (scale bar = 100 µm); (**D**) mean cell diameter and (**E**) area of eWAT; (**F**) frequency distribution of adipocyte area in eWAT among STD, HFD and HFD + K mice. Results are expressed as means ± SEM (*n* = 8). *** *p* < 0.001 versus STD mice; # *p* < 0.05 versus HFD mice.

Given that body weight gain was lower in the HFD + K group than in the HFD group after the 24-week dietary intervention, we next examined changes in WAT. As shown in Figure 3A,B, HFD feeding significantly increased epididymal white adipose tissue (eWAT) mass and the eWAT-to-body weight ratio compared with STD-fed mice. Kumquat supplementation attenuated HFD-induced adipose tissue accumulation, as evidenced by reduced eWAT weight (Figure 3A) and eWAT-to-body weight ratio (Figure 3B), relative to HFD animals. Histological examination of eWAT by H&E staining revealed marked adipocyte hypertrophy in HFD-fed mice, characterized by increased adipocyte diameter and area compared with the STD group (Figure 3C–E). Kumquat supplementation significantly reduced both adipocyte diameter and area (Figure 3C–E) and shifted the frequency distribution of adipocyte area toward smaller adipocytes (Figure 3F), suggesting improved adipose tissue morphology.

### 3.4. Changes in WAT Inflammation

**Figure 4:**
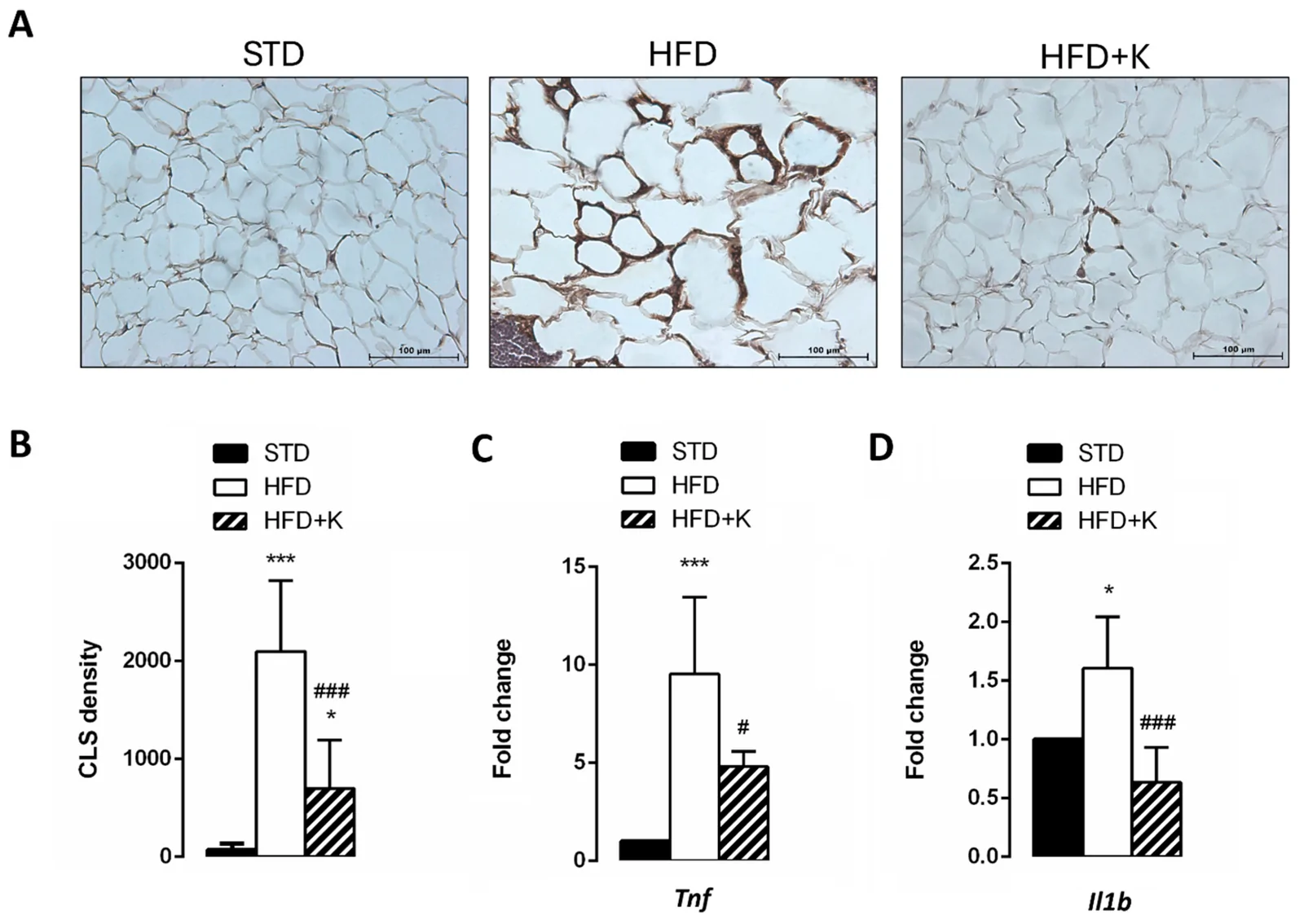
(**A**) Immunohistochemical detection of Mac-2-positive (Mac-2+) macrophages, in epididymal adipose tissues (original magnification ×200; scale bar = 100 µm); (**B**) quantification of CLS density of the immunostained section; the mRNA expression of inflammatory factors (**C**) *Tnf* and (**D**) *Il1b* in epididymal adipose tissues among STD, HFD and HFD + K mice. Results are expressed as means ± SEM (*n* = 8). * *p* < 0.05, *** *p* < 0.001 versus STD mice; # *p* < 0.05, ### *p* < 0.001 versus HFD mice.

We next investigated the effects of kumquat supplementation on inflammation in eWAT, one of the adipose depots most susceptible to obesity-associated inflammation during HFD feeding. After 24 weeks, HFD mice exhibited an increased accumulation of crown-like structures (CLSs), characterized by macrophages surrounding dead or dying adipocytes (Figure 4A,B). In contrast, kumquat supplementation significantly reduced CLS density in the eWAT of HFD + K mice (Figure 4A,B). Consistent with these histological findings, gene expression analysis revealed that the mRNA levels of the pro-inflammatory cytokines *Tnf* and *Il1b* were significantly lower in the HFD + K group than in HFD mice (Figure 4C,D).

### 3.5. Changes in the Expression of Metabolic Signalling Pathway in WAT

**Figure 5:**
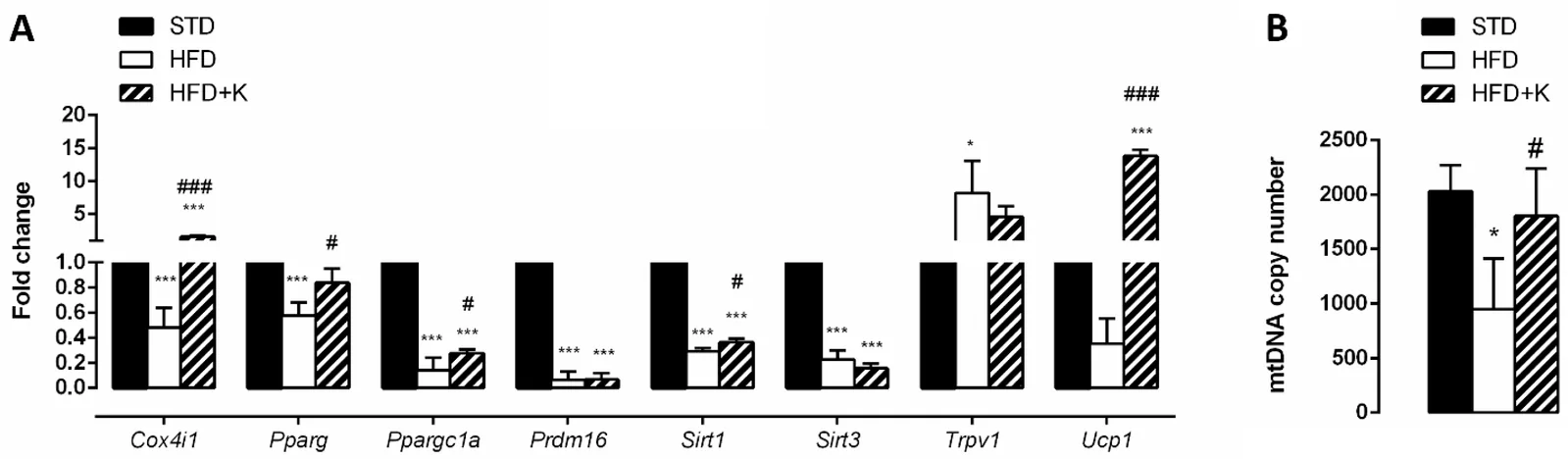
(**A**) mRNA expression of *Cox4i1*, *Pparg*, *Ppargc1a*, *Prdm16*, *Sirt1*, *Sirt3*, *Trpv1*, *Ucp1* and (**B**) Absolute mitochondrial DNA copy number, expressed as mtDNA copies per diploid cell, in eWAT among STD, HFD and HFD + K mice. Results are expressed as means ± SEM (*n* = 8). * *p* < 0.05, *** *p* < 0.001 versus STD mice; # *p* < 0.05, ### *p* < 0.001 versus HFD mice.

To gain insight into the mechanisms underlying the metabolic effects of kumquat, we next evaluated the expression of genes involved in adipose tissue browning, thermogenic activation, and mitochondrial biogenesis in eWAT. HFD feeding significantly reduced the mRNA expression of *Cox4i1*, *Pparg*, *Ppargc1a*, *Prdm16*, *Sirt1*, *Sirt3*, and *Ucp1* compared with the STD group (Figure 5A). In contrast, *Trpv1* expression was significantly increased in HFD mice relative to STD controls. Kumquat supplementation significantly upregulated the expression of *Cox4i1*, *Pparg*, *Ppargc1a*, *Sirt1*, and *Ucp1* compared with HFD mice (Figure 5A), indicating changes in the expression of genes associated with thermogenic and mitochondrial pathways in eWAT. To further assess mitochondrial-related changes, we quantified absolute mitochondrial DNA (mtDNA) copy number in eWAT. Kumquat supplementation significantly increased mtDNA copy number in HFD + K mice compared with the HFD group (Figure 5B).

## 4. Discussion

The present study shows that long-term dietary supplementation with whole kumquat is associated with reduced body weight gain and attenuation of adipose tissue dysfunction in HFD-fed mice. Specifically, kumquat supplementation reduces visceral adiposity and adipocyte hypertrophy, improves the circulating adipokine profile, attenuates adipose tissue inflammation, and modulated the expression of genes associated with mitochondrial and thermogenic pathways.

Obesity is no longer considered merely a disorder of excessive fat accumulation but rather a chronic metabolic disease characterized by alterations in adipose tissue structure and function that ultimately contribute to systemic metabolic dysfunction [26,27,28]. Indeed, accumulating evidence indicates that the metabolic consequences of obesity are more closely related to adipose tissue dysfunction than to fat mass per se. Adipocyte hypertrophy, chronic low-grade inflammation, mitochondrial impairment, and altered adipokine secretion are now recognized as key determinants of insulin resistance, ectopic lipid deposition, and cardiometabolic complications [29,30]. Consequently, preserving WAT functionality has emerged as an important target for preventing or ameliorating obesity-associated metabolic dysfunction. 

Although increasing evidence supports the beneficial effects of plant-derived bioactive compounds in the context of obesity [31], the impact of chronic kumquat consumption on adipose tissue homeostasis has remained largely unexplored. Unlike most citrus fruits, which are typically consumed after removal of the peel, kumquat is traditionally eaten whole, allowing the simultaneous intake of pulp and peel. This represents a distinctive nutritional feature, as the peel is particularly rich in flavonoids, phenolic compounds, carotenoids, dietary fibre, and essential oils [15,20,21,32] which may act synergistically to exert increased beneficial metabolic effects compared to other citrus fruits. Therefore, investigating the biological activity of the whole fruit is of particular interest, as it closely reflects human dietary consumption and preserves the natural phytochemical matrix of kumquat.

In the present study, HFD-fed mice exhibited higher body weight, increased epididymal fat mass, enlarged adipocytes, macrophage infiltration, and a dysregulated adipokine profile in comparison with STD-fed mice, confirming the well-established effects of high-fat diet feeding, as previously reported [25,33,34,35]. Long-term kumquat supplementation significantly reduced body weight gain in HFD-fed mice suggesting an anti-obesogenic effect. However, since HFD + K mice consumed significantly less food than HFD animals, the reduction in body weight gain may have been influenced, at least in part, by the lower energy intake rather than reflecting a direct metabolic effect of kumquat. Nevertheless, caloric efficiency was also significantly reduced in HFD + K mice, indicating lower body weight gain relative to the amount of energy consumed. However, in the absence of a pair-fed HFD control group, it is not possible to determine to what extent the observed effects are attributable to reduced energy intake or to metabolic effects of kumquat independent of food intake. To further investigate whether the reduced food intake observed in HFD + K mice was associated with changes in endocrine pathways involved in appetite regulation, we measured some of the major peripheral hormones regulating appetite and food intake, GLP-1, ghrelin, and leptin [36,37]. Consistent with previous reports [38], chronic HFD feeding was associated with impaired gut hormone homeostasis, characterized by reduced circulating ghrelin and lower GLP-1 concentrations. In parallel, HFD mice displayed the expected increase in circulating leptin levels, reflecting the expansion of adipose tissue and the development of obesity-associated hyperleptinemia [37]. However, long-term kumquat supplementation did not restore the circulating GLP-1 or ghrelin levels, suggesting that the reduction in food intake was not associated with normalization of these hormonal signals. In contrast, chronic kumquat supplementation significantly reduced circulating leptin concentrations, consistent with the concomitant reduction in adiposity and improvement in the adipokine profile. Nevertheless, because lower leptin levels would not be expected to suppress appetite, the concomitant reduction in food intake is unlikely to be explained by changes in leptin signalling alone. Overall, these findings suggest that the reduced food intake associated with kumquat supplementation is not readily explained by changes in the major peripheral appetite-regulating hormones examined in this study. Instead, the attenuation of body weight gain could be attributable to improvements in energy expenditure, as supported by the reduced caloric efficiency observed in HFD + K mice. 

One of the most relevant findings of this study is the reduction in adipocyte hypertrophy observed in HFD + K mice. Adipocyte size is recognized as an important indicator of metabolic dysfunction, as hypertrophic adipocytes exhibit impaired insulin responsiveness, increased lipolysis, altered adipokine secretion, and enhanced production of pro-inflammatory mediators [4,39]. In contrast, smaller adipocytes generally retain metabolic flexibility and lipid storage capacity, thereby limiting ectopic lipid deposition in peripheral organs. The shift toward smaller adipocyte populations observed in HFD + K mice therefore suggests a more favourable pattern of adipose tissue remodelling, which may be associated with the systemic metabolic improvements previously observed in the liver and brain following chronic kumquat supplementation [18,19].

The improvement in adipose tissue morphology was accompanied by significant changes in circulating metabolic hormones. Chronic Kumquat supplementation significantly increased circulating adiponectin levels while reducing leptin concentrations and, consequently, the leptin/adiponectin ratio. This ratio has emerged as a sensitive biomarker of adipose tissue dysfunction and metabolic risk, reflecting the balance between pro-inflammatory and insulin-sensitizing adipokines [40]. The increase in adiponectin is particularly relevant because this adipokine exerts insulin-sensitizing, anti-inflammatory, and mitochondria-protective effects through activation of AMPK and PPARα signalling [41]. Therefore, the improvement in the adipokine profile may contribute to the beneficial metabolic effects associated with chronic kumquat supplementation.

Chronic inflammation within white adipose tissue is considered a major driving force linking obesity to insulin resistance and metabolic disease [42]. Therefore, we evaluated the inflammatory status of eWAT following chronic kumquat supplementation. HFD feeding markedly increased CLS density and the expression of the pro-inflammatory cytokines *Tnf* and *Il1b*, as previously reported [25,34,43]. Kumquat supplementation significantly reduced CLS density and inflammatory gene expression, suggesting attenuation of obesity-associated adipose inflammation. This finding is consistent with previous evidence describing the anti-inflammatory effects of citrus polyphenols and flavonoids in obesity [44]. These anti-inflammatory effects may be related to the presence of several bioactive compounds in kumquat, including ascorbic acid and flavonoids such as eriocitrin, narirutin, and hesperidin [15,21]. These phytochemicals have been shown to inhibit NF-κB signalling, suppress the production of pro-inflammatory cytokines, and attenuate oxidative stress in experimental models of metabolic disease [45,46,47]. Moreover, D-limonene, the most abundant monoterpene among the essential oils in kumquat [20], may also contribute to the observed anti-inflammatory effects. This compound has been reported to exert antioxidant and anti-inflammatory activities through the modulation of inflammatory signalling pathways and the reduction in pro-inflammatory cytokine production, including IL-1β, IL-6, and TNF-α [9,48]. Taken together, these observations suggest that the anti-inflammatory effects associated with whole-kumquat supplementation may reflect the combined activity of its multiple bioactive components, including flavonoids and volatile compounds such as D-limonene.

We assessed whether long-term kumquat intake affects the transcriptional regulation of genes associated with mitochondrial and thermogenic pathways in eWAT of obese mice. The HFD feeding markedly reduced the expression of *Ppargc1a*, *Sirt1*, *Cox4i1*, *Pparg*, and *Ucp1*, whereas chronic kumquat supplementation significantly increased their expression, indicating modulation of molecular markers associated with these pathways. Overall, the present findings indicate that chronic kumquat supplementation may contribute to the preservation of molecular pathways associated with mitochondrial homeostasis in eWAT under HFD conditions.

It is well known that impaired mitochondrial biogenesis and oxidative metabolism contribute to adipocyte hypertrophy, chronic inflammation, altered adipokine secretion, and insulin resistance [30]. Consistent with this concept, experimental models of diet-induced obesity and obese patients exhibit reduced expression of key regulators of mitochondrial function, including *Pparg*, *Ppargc1a*, *Sirt1*, *Sirt3*, and genes involved in oxidative phosphorylation, highlighting the disruption of mitochondrial homeostasis as a major contributor to obesity-associated metabolic deterioration [49,50,51]. Within this scenario, our results indicate that long-term kumquat supplementation counteracts the HFD-induced reduction in the expression of several genes associated with mitochondrial and thermogenic pathways. In fact, kumquat increased the expression of *Pparg*, *Ppargc1a*, *Sirt1*, *Cox4i1*, and *Ucp1*, while simultaneously increasing mitochondrial DNA copy number in epididymal WAT, providing molecular evidence of adaptations associated with mitochondrial remodelling in obese adipose tissue. In particular, the simultaneous upregulation of *Sirt1* and *Ppargc1a* is noteworthy, as the SIRT1/PGC-1α axis is a key regulatory pathway associated with mitochondrial biogenesis, oxidative metabolism, and adaptive thermogenesis. Interestingly, we also observed increased *Trpv1* expression in HFD-fed mice that was not further enhanced by kumquat supplementation. TRPV1 has emerged as an important nutrient-sensitive regulator of adipose tissue metabolism, capable of modulating sympathetic activation, thermogenesis, and inflammatory signalling [52]. The increased expression observed following HFD may represent a compensatory response to chronic metabolic stress rather than a beneficial adaptation. Since kumquat did not further modify *Trpv1* expression despite modulating genes associated with thermogenic pathways, our findings suggest a possible involvement of the SIRT1–PGC-1α axis rather than changes in TRPV1 expression. Nevertheless, the precise contribution of TRPV1 warrants further investigation. Notably, although PRDM16 is widely recognized as a master regulator of brown and beige adipocyte identity [53], its gene expression remained unchanged in the kumquat-treated group despite the significant upregulation of *Ucp1*. This observation suggests that the increased *Ucp1* mRNA expression may occur through PRDM16-independent mechanisms or through modulation of PRDM16 activity at the post-transcriptional or post-translational level rather than through changes in its mRNA abundance. Indeed, UCP1 expression can also be stimulated by PPARγ and PGC-1α signalling [54].

Some limitations should also be acknowledged. The study was performed exclusively in male mice, and therefore possible sex-specific responses remain to be determined. Another limitation is the absence of a pair-fed HFD control group. Since HFD + K mice exhibited lower food intake than HFD controls, the present experimental design does not allow us to fully distinguish direct effects of kumquat supplementation from those secondary to reduced energy intake. Although the lower caloric efficiency observed in HFD + K mice suggests an altered relationship between energy intake and body weight gain, pair-feeding experiments will be necessary to establish whether the effects of kumquat on adipose tissue are independent of reduced caloric intake. In addition, although changes in gene expression and mtDNA copy number are consistent with molecular adaptations associated with mitochondrial and thermogenic pathways, protein-level analyses and direct functional measurements of mitochondrial respiration, heat production, oxygen consumption, and whole-body energy expenditure were not performed. Therefore, the observed transcriptional changes cannot be assumed to translate into corresponding alterations in protein abundance or functional activation of thermogenesis or mitochondrial biogenesis, and protein-level and functional analyses will be required to establish the functional significance of these molecular adaptations. Finally, identification of the specific phytochemical components responsible for the observed effects will require future studies employing purified fractions or metabolomic approaches.

In conclusion, the present study suggests that chronic dietary supplementation with whole kumquat attenuates obesity-associated adipose tissue dysfunction, as evidenced by reduced adipocyte hypertrophy, an improved adipokine profile, attenuated inflammation, and modulation of molecular markers associated with mitochondrial and thermogenic pathways. These findings provide new evidence that chronic whole-kumquat consumption is associated with improved adipose tissue homeostasis during diet-induced obesity.

## Figures and Tables

**Table 1:** QuantiTect Primer Assays used for quantitative real-time PCR analysis.

Gene Target	QuantiTect Primer Assay	GeneGlobe ID
*Cox4i1*	Mm_Cox4i1_1_SG QuantiTect Primer Assay	QT00108626
*Pparg*	Mm_Pparg_vb.1_SG QuantiTect Primer Assay	QT02266166
*Ppargc1a*	Mm_Ppargc1a_1_SG QuantiTect Primer Assay	QT00156303
*Prdm16*	Mm_Prdm16_1_SG QuantiTect Primer Assay	QT00148127
*Sirt1*	Mm_Sirt1_va.1_SG QuantiTect Primer Assay	QT01546083
*Sirt3*	Mm_Sirt3_1_SG QuantiTect Primer Assay	QT00147280
*Ucp1*	Mm_Ucp1_1_SG QuantiTect Primer Assay	QT00097300
*Trpv1*	Mm_Trpv1_1_SG QuantiTect Primer Assay	QT00180782
*Actb*	Mm_Actb_1_SG QuantiTect Primer Assay	QT00095242

## Data Availability

The original contributions presented in this study are included in the article/Supplementary Materials. Further inquiries can be directed to the corresponding author.

## Supplementary Materials

The following supporting information can be downloaded at: https://www.mdpi.com/article/10.3390/cimb48101048/s1.

## Abbreviations

The following abbreviations are used in this manuscript:

AMPK	AMP-activated protein kinase
CLSs	crown-like structures
*Cox4i1*	Cytochrome c oxidase subunit 4 isoform 1
eWAT	epididymal white adipose tissue
GLP-1	glucagon-like peptide-1
H&E	Haematoxylin and Eosin
HFD	high-fat diet
HFD + K	HFD supplemented with lyophilized whole kumquat
*Il1b*	Interleukin-1
IL-6	interleukin-6
MASLD	metabolic dysfunction-associated steatotic liver disease
MCP-1	monocyte chemoattractant protein-1
mtDNA	mitochondrial DNA
PGC-1α	Peroxisome proliferator-activated receptor gamma coactivator 1-alpha
*Pparg*	Peroxisome proliferator-activated receptor gamma
*Ppargc1a*	Peroxisome proliferator-activated receptor gamma coactivator 1-alpha
*Prdm16*	PR domain containing 16
SEM	standard error of the mean
*Sirt1*	Sirtuin 1
*Sirt3*	Sirtuin 3
STD	Standard diet
T2DM	Type 2 Diabetes Mellitus
TNF-α	Tumour necrosis factor-α
*Trpv1*	Transient receptor potential vanilloid 1
*Ucp1*	Uncoupling protein 1
WAT	white adipose tissue
