# Supplementary material for: Adipose Tissue Adaptations to Chronic Kumquat Consumption in High-Fat Diet-Induced Obese Mice
Source: Curr Issues Mol Biol. 2026 Oct 10;48(10):1048. doi: 10.3390/cimb48101048 (PMC13703289; doi:10.3390/cimb48101048)
Supplement: Supplementary file 1 [file cimb-48-01048-s001.zip › Supplementary Figure S1.pdf]

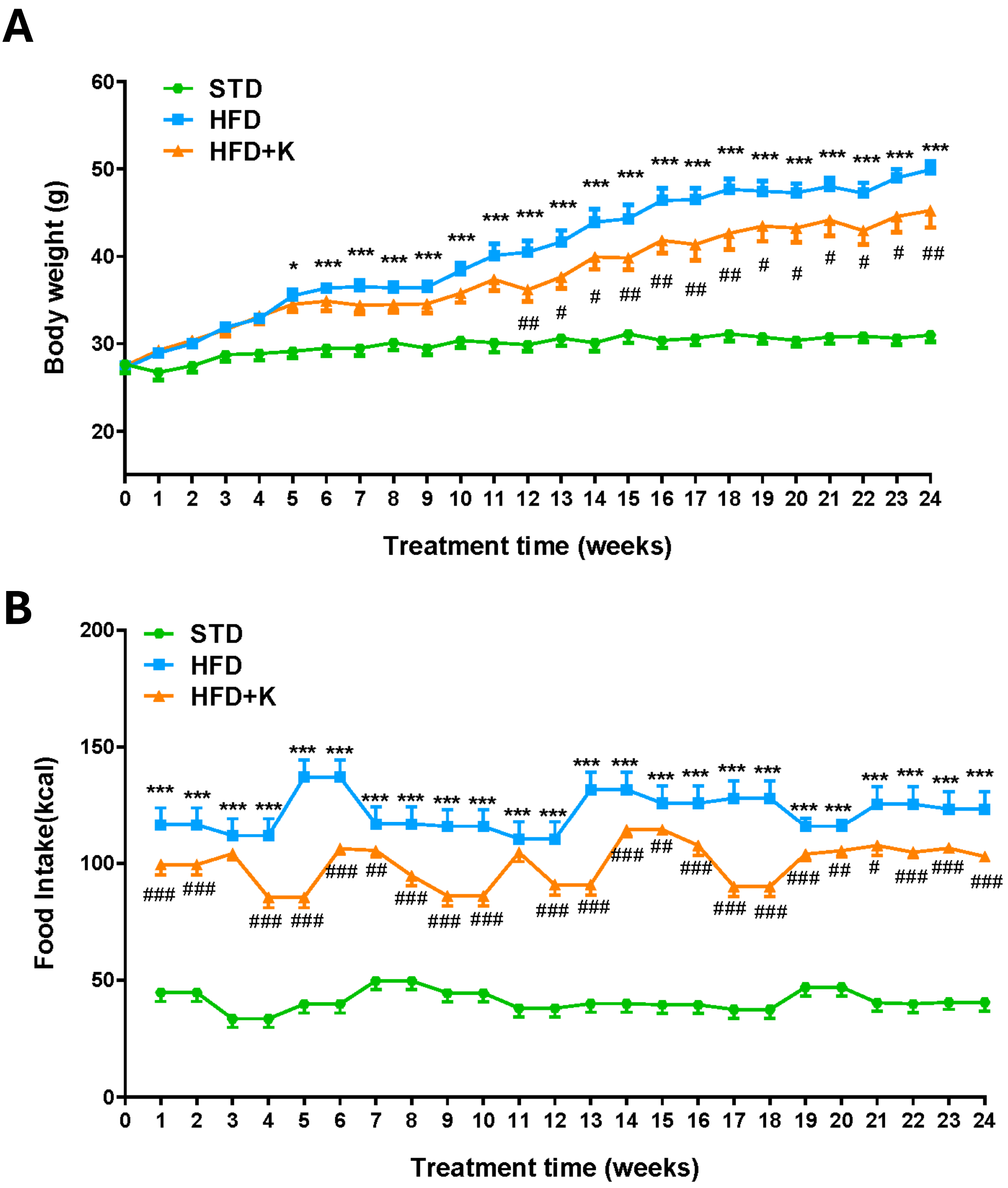

Supplementary Figure S1. A) Body weight and B) food intake, measured weekly throughout the experimental period (24 weeks) in mice fed standard diet (STD), high-fat diet (HFD) and high-fat diet supplemented with kumquat (HFD+K). Results are expressed as means ± SEM (n = 8). Statistical significance was evaluated using two-way analysis of variance (two-way ANOVA). * $p < 0.05$, *** $p < 0.001$ versus STD mice; # $p < 0.05$, ## $p < 0.01$, ### $p < 0.001$ versus HFD mice.
