# Supplementary material for: Adipose Tissue Adaptations to Chronic Kumquat Consumption in High-Fat Diet-Induced Obese Mice
Source: Curr Issues Mol Biol. 2026 Oct 10;48(10):1048. doi: 10.3390/cimb48101048 (PMC13703289; doi:10.3390/cimb48101048)
Supplement: Supplementary file 1 [file cimb-48-01048-s001.zip › Table S1.pdf]

Table S1. Formulation and energy density of the HFD and kumquat-supplemented HFD

Dietary component	HFD (g/kg diet)	HFD+K (g/kg diet)
Casein, lactic, 30 mesh	200.0	200.0
L-Cystine	4.0	4.0
Maltodextrin-0032	160.0	160.0
Sucrose	**90.0**	**40.0**
Cellulose (Arbocel)	65.5	65.5
Lard	220.0	220.0
Soybean oil	30.0	30.0
Mineral mix AIN-93G-MX-PF2348	48.0	48.0
Choline bitartrate	3.0	3.0
Vitamin mix AIN-93-VX-PF2439	21.0	21.0
Calcium phosphate, dibasic	3.4	3.4
**Lyophilized whole kumquat**	—	**50.0**
**Energy density (kcal/g)**	**6.00**	**6.00**
