# Supplementary material for: Adipose Tissue Adaptations to Chronic Kumquat Consumption in High-Fat Diet-Induced Obese Mice
Source: Curr Issues Mol Biol. 2026 Oct 10;48(10):1048. doi: 10.3390/cimb48101048 (PMC13703289; doi:10.3390/cimb48101048)
Supplement: Supplementary file 1 [file cimb-48-01048-s001.zip › Table_S2_.pdf]

Table S2. Nutritional and phytochemical composition of *Fortunella japonica*.

Category	Components	Reported content	Matrix
	**Energy**	71 kcal/100 g	
**Macronutrients**			Whole fruit
	Carbohydrates	15.90 g/100 g	
	Total fat	0.86 g/100 g	
	Protein	1.88 g/100 g	
	Ash	0.61 ± 0.09%	
**Micronutrients**			Whole fruit
**Minerals**			
	Iron	2.04 mg/100 g	
	Manganese	0.47 mg/100 g	
	Copper	0.46 mg/100 g	
	Magnesium	35.70 mg/100 g	
	Potassium	156.34 mg/100 g	
	Calcium	181.20 mg/100 g	
	Phosphorus	17.75 mg/100 g	
	Zinc	Trace amount	
**Vitamin**			
	Vitamin C (ascorbic acid)	55.29 mg/100 g	
**Carotenoids**			Whole fruit
	β-Carotene	0.33 mg/100 g	
	β-Cryptoxanthin	0.26 mg/100 g	
	Lutein	0.44 mg/100 g	
	Zeaxanthin	0.24 mg/100 g	
**Flavonoids**			Whole fruit
	Narirutin	107 mg/100 g	
	Rhoifolin	36.66 mg/100 g	
	Rutin	4.3 mg/100 g	
	Naringin	2.79 mg/100 g	
	Quercetin	30.8 mg/100 g	
	Kaempferol	23.5 mg/100 g	
	Diosmin	69.9 µg/100 g	
	Sinensetin	164 µg/100 g	
	Hesperidin	36.6 µg/100 g	
**Phenolic acids**			Whole fruit
	Chlorogenic acid	5 mg/100 g	
	Ferulic acid	0.492 mg/100 g	
	Sinapic acid	1.15 mg/100 g	
	p-coumaric acid	2.21 mg/100 g	
**Volatile compounds**			Peel
	Limonene	93.73%	
	Myrcene	1.84%	
	Ethyl acetate	1.13%	
**C-glycosyl flavonoids**			Peel
	3’,5’-Di-C-β-glucopyranosylphloretin (DGPP)	2082 mg/100 g	
	Acacetin 8-C-neohesperidoside (margaritene)	372 mg/100 g	
	Fortunellin	234 mg/100 g	
	Acacetin-6-C-neohesperidoside (isomargaritene)	205 mg/100 g	
	Apigenin-8-C-neohesperidoside	56.5 mg/100 g	
	Poncirin	33 mg/100 g	
	Rhoifolin	7.4 mg/100 g	

Sources: [25, 28, 52-54]
